# Green Synthesis of Iron Nanoparticles and Their Environmental Applications and Implications

**DOI:** 10.3390/nano6110209

**Published:** 2016-11-12

**Authors:** Sadia Saif, Arifa Tahir, Yongsheng Chen

**Affiliations:** 1 Department of Environmental Science, Lahore College for Women University, Lahore 54000, Pakistan; arifa.tahir@gmail.com; 2 School of Civil and Environmental Engineering, Georgia Institute of Technology, Atlanta, GA 30332, USA

**Keywords:** iron nanomaterials, sustainable green nanotechnology, environmental pollution, environmental toxicology

## Abstract

Recent advances in nanoscience and nanotechnology have also led to the development of novel nanomaterials, which ultimately increase potential health and environmental hazards. Interest in developing environmentally benign procedures for the synthesis of metallic nanoparticles has been increased. The purpose is to minimize the negative impacts of synthetic procedures, their accompanying chemicals and derivative compounds. The exploitation of different biomaterials for the synthesis of nanoparticles is considered a valuable approach in green nanotechnology. Biological resources such as bacteria, algae fungi and plants have been used for the production of low-cost, energy-efficient, and nontoxic environmental friendly metallic nanoparticles. This review provides an overview of various reports of green synthesised zero valent metallic iron (ZVMI) and iron oxide (Fe_2_O_3_/Fe_3_O_4_) nanoparticles (NPs) and highlights their substantial applications in environmental pollution control. This review also summarizes the ecotoxicological impacts of green synthesised iron nanoparticles opposed to non-green synthesised iron nanoparticles.

## 1. Introduction

Nanotechnology is the ability to measure, see, manipulate and manufacture things on an atomic or molecular scale, usually between one and 100 nanometres. These tiny products also have a large surface area to volume ratio, which is their most important feature responsible for the widespread use of nanomaterials in mechanics, optics, electronics, biotechnology, microbiology, environmental remediation, medicine, numerous engineering fields and material science [1]. Different protocols have been designed for the production of metallic nanoparticles. Currently, two main approaches are used to synthesize nanoparticles, referred to as the top-down and bottom-up approaches. Briefly, in the top-down approach, nanoparticles are produced by size reduction of bulk material by lithographic techniques and by mechanical techniques such as machining and grinding, etc., while, in bottom-up approach, small building blocks are assembled into a larger structure, e.g., chemical synthesis [2]. However, the most acceptable and effective approach for nanoparticle preparation is the bottom-up approach, where a nanoparticle is “grown” from simpler molecules known as reaction precursors. In this way, it is likely possible to control the size and shape of the nanoparticle depending on the subsequent application through variation in precursor concentrations and reaction conditions (temperature, pH, etc.) [3].

**Figure 1:**
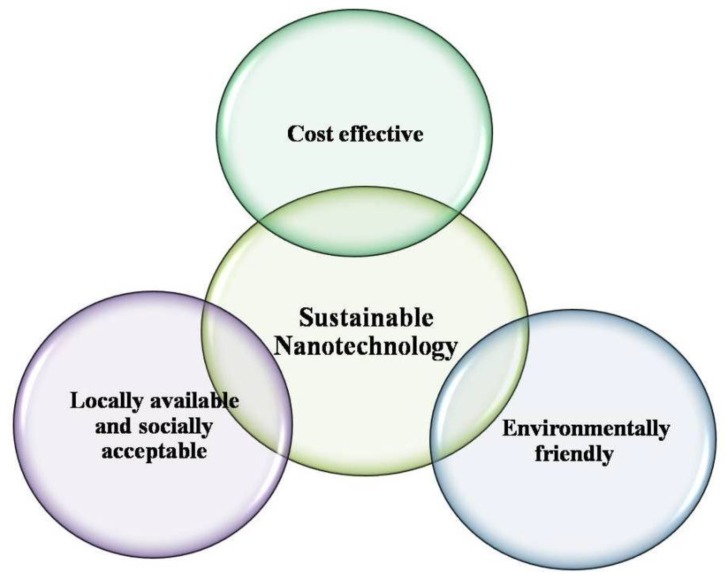
Sustainable green nanotechnology.

Physical and chemical methods are being used extensively for production of metal and metal oxide nanoparticles. However, this production requires the use of very reactive and toxic reducing agents such as sodium borohydride and hydrazine hydrate, which cause undesired detrimental impacts on the environment, plant and animal life it supports. Researchers continue efforts to develop facile, effective and reliable green chemistry processes for the production of nanomaterials. Various organisms act as clean, eco-friendly and sustainable precursors to produce the stable and well functionalised nanoparticles. These may include bacteria, actinomycetes, fungi, yeast, viruses, etc. [4,5]. Thus, it is vitally important to explore a more reliable and sustainable process for the synthesis of nanomaterials. Economic viability, environmental sustainability, and social adaptability as well as the availability of local resources are a matter of concern in the production of nanomaterials (Figure 1). In order to keep the prices of the final finished nanotechnology-based products affordable to consumers, industries must maintain a delicate balance between environmentally sound green processes and their sustainability. The green nanotechnology-based production processes operate under green conditions without the intervention of toxic chemicals.

Many recent studies have indicated the potential of iron nanoparticles (NPs) for environmental remediation. Nanoscale materials such as nanoadsorbents, nanocatalysts, nanofiltration, and nanobiocides such as metal and metal oxide nanoparticles are currently being employed for remediation of water and wastewater pollutants. Among these metallic nanoparticles, iron nanoparticles (FeNPs) have promising advantages that can combat environmental pollution. The interest in nanoscale zero-valent iron (nZVI) in environmental remediation is increasing due to the reactivity of nanoscale iron having a large surface area to volume ratio [6,7]. The production of iron nanomaterials, such as metallic iron and oxide of iron via a more convenient greener route, is a great step forward in the development of nanomaterials. This review highlights the significance of biogenic approaches and the role of biocompatible green materials in technological and economically feasible process and practices. It also summarizes the quest for an environmentally sustainable synthesis process of iron nanomaterials for their application to the field of environmental sustainability.

## 2. Green Routes for the Synthesis of Metallic Iron Nanoparticles

### 2.1. Synthesis by Biocompatible Green Reagents

***Biopolymers*:** Research has been performed to utilize non-toxic synthetic biocompatible materials for the synthesis, as well as for stabilisation of magnetic nanoparticles polymer composites (Table 1). In this scenario, He et al. [8] used water soluble starch for stabilisation of bimetallic Fe/Pd nanoparticles. Starch is a hydrophilic polymer, which consists of ~20% amylose; in this study, it was found that starch plays a significant role in dispersion and stabilisation of iron nanoparticles. In another study, synthesis of magnetite (Fe_3_O_4_) nanoparticles was achieved by a biopolymer sodium alginate by redox-based hydrothermal method using FeCl_3_·6H_2_O and urea as the starting materials. Sodium alginate fabricated nanoparticles showed uniform and spherical morphology with mean diameter of 27.2 nm [9]. Well dispersed magnetite (Fe_3_O_4_) agar nanocomposite was prepared by co-precipitation of Fe(III) and Fe(II) ions for the first time by Jegan et al. [10].

***Ascorbic acid*:** Synthesis of iron nanoparticles using ascorbic acid (Vitamin C) have been studied by Nadagouda et al. [11]. Core-shell Fe and Cu nanoparticles have been produced by using aqueous ascorbic acid (Vitamin C) which reduced the transition metal salts into their respective nanostructures. Likewise Savasari et al. [12] used ascorbic acid to produce stabilised zero valent iron nanoparticles assembled in a chain in which individual particles were round in shape with a diameter of 20 to 75 nm. Moreover, ascorbic acid has been used as functionalizing and stabilizing agent for nanoparticles. In one study, superparamagnetic iron oxide nanoparticles were coated and further functionalised by using ascorbic acid (Vitamin C) to form a stable dispersion for medical application. The transmission electron microscope (TEM) image of the coated nanoparticles revealed that particles were spherical in shape with an average particle size of 5 nm [13].

***Amino acids*:** Krishna et al. [14] carried out research to produce amine functionalised magnetite nanoparticles by the wet chemical co-precipitation method. A highly crystalline magnetite phase was obtained by (in-situ) functionalisation with l-lysine amino acid. Similarly, Siskova et al. [15] used different amino acids such as l-glutamic acid, l-glutamine, l-arginine and l-cysteine to synthesize zero valent iron and studied the effect of pH on zero valet iron generation.

***Haemoglobin and myoglobin*:** In one study, Sayyad et al. [16] reported the one-pot synthesis of iron nanoparticles (Fe NPs) from naturally available Fe-containing bio-precursors, i.e., haemoglobin and myoglobin. A single-phase chemical reduction reaction produced the stable iron nanoparticles at room temperature. The size distribution of the synthesised particles fall into the narrow 2–5 nm range and the particles were observed to be crystalline. This strategy can be an important valuable engineering approach for fabrication of bio-conjugated nanoparticle for biological applications.

***Sugar and Glucose:*** Lu et al. [17] prepared polycrystalline Fe_3_O_4_ nanoparticles using d-glucose as the reducing agent and gluconic acid (the oxidative product of glucose) as a stabilizer and dispersant. A detailed characterisation was performed to reveal the Fe_3_O_4_ nanoparticle structures. Transmission electron microscopy (TEM) results exhibited that Fe_3_O_4_ nanoparticles have a roughly spherical shape and their average size is about 12.5 nm. Sun et al. [18] synthesised magnetite (Fe_3_O_4_) nanoparticles coated with glucose and gluconic acid via a facile hydrothermal reduction approach utilizing a single iron source i.e., FeCl_3_. In hydrothermal reduction process sucrose decomposed into reducing species, causing partial reduction of the Fe^3+^ ions to Fe^2+^ ions for the formation of Fe_3_O_4_, furthermore, capped the nanoparticles to change the surface properties and enable the formation of nanoparticles.

In a recent study, Yan et al. [19] utilised wood-derived sugar to synthesize carbon encapsulated iron nanoparticles under hydrothermal carbonisation conditions. Detailed characterisation was done on nanospheres, which were around 100–150 nm in diameter with an iron core diameter of 10–25 nm. The catalytic effect of carbon-encapsulated iron nanoparticles to convert the syngas into liquid hydrocarbons was evaluated by Yan et al. [19].

***Synthetic tannic and Gallic acid*:** Herrera-Becerra et al. [20] reported the synthesis of iron oxide nanoparticles by powder tannic acid. Highly crystalline and monodisperse iron oxide (Fe_2_O_3_) nanoparticles were prepared by aqueous suspension of tannic acid under ultrasonic treatment at a controlled pH of 10. The high resolution transmission electron microscopy (HR-TEM) results showed that the biosynthesised NPs were spherical in shape with sizes smaller than 10 nm [20]. Dorniani et al. [21] produced magnetic iron oxide nanoparticles by the sonochemical method and consequently coated the NPs with chitosan and Gallic acid to create a core-shell structure. X-ray diffraction (XRD) demonstrated that the magnetic nanoparticles were pure Fe_3_O_4_ with a cubic inverse spinel morphology with an average diameter of 13 nm.

### 2.2. Synthesis by Microorganisms

***Bacteria:*** Iron reducing bacteria are commonly used in synthesis of iron nanomaterials. Bharde et al. [22] synthesised spherical iron oxide nanoparticles using *Actinobacter* sp. under aerobic conditions. In another study, maghemite (γ-Fe_2_O_3_) and greigite (Fe_3_S_4_) were synthesised using the same species of bacterium by altering the iron precursor. Here, *Actinobacter* sp. was found to be capable of extracellular synthesis of magnetic nanoparticles when exposed to the aqueous solution of ferric salts under aerobic conditions for 48–72 h. The formation of iron oxide nanoparticles was indicated by changed in colour of reaction, medium to dark brown and further characterised by TEM, XRD, FTIR, magnetic measurements, etc. Bacterial synthesis of magnetic particles is a complex phenomenon, and synthesis involves the enzyme iron reductase produced by *Actinobacer* sp. in presence of iron salt. Iron reductase, reduce the Fe^3+^ into Fe^2+^, extracellularly for formation magnetic particles. In this study, Fe^3+^ reductase activity was confirmed by Ferrisiderophore reductase assay which indicated that extracellular iron reductase was synthesised in the presence of excess iron salt [23]. Moon et al. [24] prepared magnetite nanoparticles under anaerobic conditions using a thermophilic strain, *Thermoanaerobacter* sp. and FeOOH as the precursor. Extracellular magnetites exhibited good mono-dispersity with a mean diameter of 13.1 nm when analysed under transmission electron microscopy (TEM). Extracellular biosynthesis of Fe_3_O_4_ nanoparticles was performed using *Bacillus subtilis* strains isolated from rhizosphere soil. The synthesised nanoparticles were spherical in shape and diameter in the range of 60–80 nm [25]. Elcey et al. [26] used the *Thiobacillus thioparus* bacterial strain isolated from iron ore mining sites. The magnetosomes had magnetic characteristics as purified particles synthesised by isolated bacterial strains with a protein coating as evidenced by the stained polyacrylamide gel.

***Fungi*:** Different sizes of magnetic particles may be produced extracellularly by exploiting the fungi, such as *Fusarium oxysporum* and *Verticillium* sp., with mixtures of ferric and ferrous salts at room temperature. Cationic proteins secreted by the fungi cause an extracellular hydrolysis of the anionic iron complexes. Consequently, leads to formation of crystalline magnetite particles that exhibit a ferrimagnetic transition signature with insignificant amount of spontaneous magnetisation at low temperature [27]. Kaul et al. [28] tested five different species of fungi, *P. chlamydosporium*, *A. fumigates*, *A. wentii*, *C. lunata* and *C. globosum*, and two bacteria, *A. faecalis* and *B. coagulans*, for the production of iron nanoparticles [29]. Another group of researchers, Mohamed et al. [30], used *Alternaria alternata* fungus for production of Fe NPs, which has been characterised by various spectroscopic techniques. The nanoparticles were found to be 9 ± 3 nm having cubic shape. These nanoparticles exhibited antibacterial activity against *B. subtilis*, *E. coli*, *S. aureus* and *P. aeruginosa*.

***Algae*:** Mahdavi et al. [31] worked on biosynthesis of iron oxide nanoparticles (Fe_3_O_4_ NPs) by reduction of ferric chloride solution with the macroalgae, brown seaweed (*Sargassum muticum*) extract. The water extract of brown seaweed contains sulphated polysaccharides, which Mahdavi et al. used as a main factor in the reduction of iron salt. The rapid reaction was completed in one step by changing the solution colour from yellow to dark brown. The average particle diameter was 18 ± 4 nm determined by TEM. X-ray diffraction (XRD) showed that the nanoparticles were crystalline in nature, with a cubic shape. Subramaniyam et al. [32] employed soil micro algae, *Chlorococcum* sp., with an iron chloride precursor to synthesize the spherical-shaped nanoiron ranging in size from 20–50 nm. The surface of microalagl cell contained nanoiron, not only localised inside as well as outside the cell as revealed by TEM. It was suggested that biomolecules such as carbonyl and amine from polysaccharides and glycoproteins present in algal cell were involved in synthesis of nanoiron and confirmed by FTIR analysis. Reports on biosynthesis of iron nanoparticles from microorganisms have been summarized in Table 2.

### 2.3. Synthesis of Iron Nanoparticles from Plant Biomaterials

Unfortunately, the production of nanomaterial from microorganisms is less monodispersed and the rate of synthesis is slow compared to plant-based synthesis [33]. According to Kalaiarasi et al. [34], green synthesis of metallic nanoparticles by different plant parts such as the leaf, stem, seed and root is the simplest, most cost effective and reproducible approach. Plants certainly produce more stable metal nanoparticles and have proved to be the best candidates for fast and large-scale synthesis as compared to microorganisms [35]. The preference for plants and their derivatives in nanomaterial production lies in the plants’ natural composition of different organic reducing compounds, which easily adapt to the synthesis of nanoparticles [36]. Different herbs and plant sources occlude higher antioxidants that are available as phytochemical constituents in seeds, fruits, leaves and stems. Therefore, the utility of plant-based phytochemicals in the overall synthesis and architecture of nanoparticles creates an important symbiosis between natural/plant sciences and nanotechnology. This association gives a characteristically green approach to nanotechnology, referred to as green nanotechnology. These production processes can be carried out without significant environmental pollution, thereby setting new standards in highly sustainable and economically viable clean and green technologies [37].

***Synthesis by leaf extract:*** The green synthesis of iron nanoparticles using various plant extracts has been reported by many researchers. Biosynthesis of iron nanoparticles (Fe NPs) has been mainly performed using extract of green tea which is a cheap and local resource. Hoag et al. [38] synthesised nZVI utilizing green tea (*Camellia sinensis*) extract containing a range of polyphenols. Without the addition of any surfactant or polymer, the stable nanoparticles were obtained at room temperature. Polyphenols in plant act as both a reducing agent and a capping agent, resulting in stable green nanoscale zero-valent iron particles with unique properties. Green tea (20 g/L) was used for preparation of extract. A solution of 0.1 M FeCl_3_ was added to (20 g/L) green tea extract in a 2:1 volume ratio resulting in spherical nanoparticles with diameter of 5–10 nm. In another study, Shahwan et al. adopted the same procedure for synthesis of iron nanoparticles with little modification. They used the 0.10 M iron chloride solution to green tea in 2:3 volume ratios. Following this, 1.0 M NaOH solution was added until the pH was 6.0 and the formation of nanoparticles was marked by the appearance of intense black precipitate. The iron particles were harvested by evaporating water from the solution. The obtained nanoparticles (40–60 nm) were then employed as a catalyst for the degradation of methylene blue and methyl orange dyes [39]. Moreover, Markova et al. [40] prepared the iron(II, III)-polyphenol complex nanoparticles with a diameter of 70 nm-sized by adding Fe(NO_3_)_3_·9H_2_O to the green tea extract. Fe-based nanoparticles were prepared by introducing 0.5 M Fe(NO_3_)_3_·9H_2_O into green tea extract in a 1:5 volume ratio under nitrogen atmosphere. Researcher’s produced zero valent iron and iron(II, III) polyphenol complex nanoparticles by utilizing green tea extract in different studies. Hence, production of nano iron with different size and properties are due to change in synthesis procedure, and most important ratio of extract to salt. Similar findings were found in study of Nadagouda et al. [41], they evaluated the effect of extract concentration on size of iron nanoparticles. Nanoscale zero valent iron (nZVI) synthesis was done at room temperature using different volumes of tea extract and Fe(NO_3_)_3_ solution. It was found that size and morphology of particles could be change by changing the concentration of extract as well as iron salt.

Machado et al. [42] evaluated the feasibility of several tree leaves for production of nZVI. In addition, the antioxidant capacity of leaf extracts was also estimated. The results reveal that dried leaves produce extracts with higher antioxidant capacities than non-dried leaves. Leaves of oak, pomegranate and green tea produced the richest extracts, and TEM analysis confirmed that nZVIs (d = 10–20 nm) can be produced utilizing these plant resources. Use of water as the solvent for preparation of the extract is considered the cheapest and greenest method for production of nanoparticles. In another study, Pattanayak and Nayak [43] used a low-cost reductant for synthesizing nanoscale zero-valent iron (nZVI) by *Azadirachta indica* (neem) leaves extract under atmospheric conditions. The UV-Vis spectroscopy of synthesised iron nanoparticles were in the range of 216–265 nm. The size of spherical iron nanoparticles was predominantly found within the range of 50–100 nm.

Wang [44] synthesised stable iron-polyphenol complex nanoparticles (Fe-P NPs) using leaf extract of eucalyptus. Similarly in another study Wang et al. [45] utilised three different plants i.e., *Eucalyptus tereticornis*, *Melaleuca nesophila* and *Rosemarinus officinalis* to produce iron ions polyphenols complex nanoparticles (Fe-P NPs) ranging in sizes from 50 to 80 nm were. Luo et al. [46] produced Fe NP with an average size of 60 nm by utilizing methanolic grape leaf extract. Gas chromatography-mass spectrometry (GC-MS) analysis confirmed the presence of biomolecules including phytols, terpenoids, and antioxidants which involved in synthesis of nanoparticles.

Plants materials are capable of synthesize crystalline magnetite nanoparticles. Crystalline monodisperse magnetite (Fe_3_O_4_) nanoparticles were synthesised by the carob leaf in a one-step reaction [47]. An aqueous solution of ferric chloride hexahydrate and ferrous chloride tetra hydrate (2/1 molar ratio) was mixed, and magnetite nanoparticles with an average diameter of 8 nm were obtained. The Fourier transform infrared (FTIR) spectra of carob leaf extract showed NH stretching and OH overlapping of the stretching vibration band attributed to water and carob leaf extract molecules. Pattanayak and Nayak [48] exploited the different plant resources such as mango leaves, green tea leaves, rose leaves, oregano leaves and curry leaves for production of metallic iron nanoparticles. Remarkable changes in colour and pH were observed during the reduction of iron salt by extracts. Such rapidly processed plant-mediated iron metallic nanoparticles is an alternative to chemical synthesis protocols and can serve as a low cost reductant for synthesizing iron nanoparticles. Rapid synthesis of crystalline iron oxide nanoparticles (Fe_3_O_4_) was performed by reduction of ferric chloride (FeCl_3_) with leaf extract of *Tridax procumbens*. The water extract of *T. procumbens* contains water soluble carbohydrate compounds. Carbohydrates containing aldehyde groups may reduce the Fe^3+^ of ferric chloride to Fe_3_O_4_ nanoparticles [49].

Fe^0^/Fe_3_O_4_ nanoparticles were successfully synthesised using pomegranate (*Punica granatum*) leaf extract by Rao et al. [50]. Leaf extract of pomegranate was prepared in water in a 1:10 ratio *w*/*v*. Optimum synthesis was done by adding the extract (1.2 mL) to 6 mL iron salts mixtures (mixture of 0.2 M ammonium ferrous sulphate ((NH_4_)_2_Fe(SO_4_)_2_·6H_2_O) and 0.2 M ammonium ferric sulphate (NH_4_Fe(SO4)_2_·12H_2_O)) in a 1:2 ratio. The reaction was maintained for 30 min at 30 °C, under stirring and for conversion of excess iron species into Fe_3_O_4_, 1 N NaOH was added. The product was separated out by centrifugation at 6000× *g* for 15 min. These nanoparticles were utilised for modification of two strains (NCIM 3589 and NCIM 3590) of heat-killed yeast cells *Yarrowia lipolytica*, which were further employed as biosorbents to remove hexavalent chromium. The biocomposites showed the presence of Fe^0^/Fe_3_O_4_ when analysed by Mössbauer spectroscopy. The XRD profiles of the magnetic precipitate could be indexed to magnetite while SEM images showed the uniform distribution of iron nanoparticles on surface of yeast cells.

Makarov et al. [51] reported the synthesis of iron oxide nanoparticles using aqueous extract of *Hordeum vulgare* and *Rumex acetosa. Hordeum vulgare* produced the amorphous iron oxide (Fe_3_O_4_) nanoparticles with a particle size up to 30 nm. The role of pH was considerable in the stability of iron nanoparticles. The authors of this paper found that the stability of *H. vulgare* synthesised iron nanoparticles was increased by adding 40 mM of citrate buffer with pH 3.0. Similarly, amorphous iron nanoparticles with a diameter of 10–40 nm was produced by extract of *Rumex acetosa. R. acetosa* extract synthesised iron nanoparticles were highly stable due to low pH (pH = 3.7) as compared to *H. vulgare* (pH 5.8).

In recent study, Prasad et al. [52] produced iron(III) oxide nanocrystals with leaf extract of Garlic Vine and FeSO_4_·7H_2_O. The bio-precipitation was accelerated by adding a few drops of 1 M NaOH to obtain pH 6. The reaction resulted in formation of β-Fe_2_O_3_ of nanocrystals with size of 18.22 nm. According to XRD results, iron predominantly occupying the octahedral in iron(III) oxide nanocrystals. The band gap energy 2.84 eV endorsed the semiconducting transition. Furthermore, thermogravimetric analysis (TGA) measurements showed the organic coating over the surface of nanoparticles which confirmed that biomolecules stabilised the nanoparticles. Furthermore, at above temperature 500 °C, β-Fe_2_O_3_ sample undergoes to a complete phase transformation of meta-stable β-Fe_2_O_3_ to stable α-Fe_2_O_3_.

***Fruit extract:*** Some researchers use fruits for synthesis of iron nanomaterials. Mohan Kumar et al. [53] synthesised palladium and iron NPs using aqueous fruit extract of *Terminalia chebula*. Redox potential of polyphenolic rich *T. chebula* aqueous extract was 0.63 V vs. SCE (saturated calomel electrode) by cyclic voltammetry. Such a reduction helps to reduce the iron precursors to iron NPs. Remarkable stable iron nanoparticles were synthesised via simultaneous reduction of FeSO_4_·7H_2_O solution by *T. chebula* extract containing complexation of polyphenols. A 5:1 ratio of extract to metal salt solution was used and solid product was separated out by centrifugation followed by ethanolic washing. X-ray diffraction (XRD) and transmission electron microscope (TEM) analyses revealed that amorphous iron NPs were within a size of less than 80 nm.

In another study Kumar et al. [54] synthesised Fe_3_O_4_ nanoparticles by the fruit extract of *Passiflora tripartitavar* mollissima and studied their catalytic effect on the synthesis of 2-arylbenzimidazole under room temperature. Using aqueous extract of *Passiflora tripartitavar*, mollissima fruit spherical iron oxide nanoparticles of 22.3 ± 3 nm size were synthesised. The synthesised nanocatalyst is highly active for the synthesis of biologically significant 2-arylbenzimidazoles. Benzimidazole moiety is a structural isostere of naturally occurring nucleotides; hence, it has been useful in creating intermediates in the development of molecules for pharmaceutical and biological purposes. The one-pot synthesis of 2-arylbenzimidazole derivatives using Fe_3_O_4_ nanoparticles is environmentally benign, selective, and easy to manipulate. Additionally, the Fe_3_O_4_ nanoparticles as a heterogeneous catalyst could be reused five times for fresh reactions with a slight change in reactivity.

***Seed extract:*** Seed extract of *Syzygium cumini* was used as a reducing agent and sodium acetate as an electrostatic stabilizing agent for the synthesis of iron oxide nanoparticles by Venkateswarlu et al. [55]. The XRD study reveals that the synthesised spherical magnetic nanoparticles (SMNPs) have inverse spinel face-centred cubic structure 9–20 nm in diameter as shown by TEM. The presence of polyphenols, flavonoids, and other biomolecules in the *S. cumini* seed was confirmed by Fourier transform infrared (FTIR) spectroscopy technique. The Brunauer–Emmett–Teller (BET) surface area of the Fe_3_O_4_ particles was found to be 3.517 m^2^/g, and the particles were classified as mesoporous. The average pore size for the Fe_3_O_4_ was determined according to the single-point adsorption total volume at a relative pressure *P/P_O_* = 0.9905 cm^3^/g. By virtue of this property, the as-synthesised nanoparticles can be used in the field of environmental remediation for the removal of toxic metals and dyes.

### 2.4. Other Plant Materials

***Alfalfa biomass*:** Beccera and his collaborators used a green chemistry method to obtain biosynthesised iron oxide nanoparticles with sizes of less than 5 nm. *Medicago sativa* (alfalfa) biomass represented the first time iron oxide nanoparticles were produced). Milled powder of *Medicago sativa* was introduced to the salt solution of ferrous ammonium sulphate, and the effect of pH conditions was determined during the synthesis. The role of pH was determined as a size-limiting parameter for iron nanoparticle synthesis. Becerra et al. [56] found the optimum pH to obtain nanoparticles of size less than 10 nm (pH = 10). In the second study, more emphasis was placed on advanced characterisation techniques to electron microscopy-based characterisation of the above mentioned iron oxide nanoparticles. Under optimal conditions (pH = 10) aggregates of 1–10 nm were found. Often when nanoparticles were immersed in the alfalfa biomass that served as a base, the observation of nanoparticles became difficult, especially for those of less than 10 nm. Based on highly advanced techniques like the high angle annular dark field (HAADF), *Z* contrast was used to locate the nanoparticles in alfalfa biomass. Energy dispersive spectroscopy (EDS) and high resolution transmission electron microscopy (HR-TEM) were used for further characterisation of the synthesised nanoparticles [57].

***Sorghums bran:*** Njagi et al. [58] explored the diverse phenolic compounds of sorghum bran for the synthesis of iron metallic nanoparticles. Reaction was carried out at room temperature for 1 h by adding 0.1 M FeCl_3_ solution to the sorghum bran extract in a 2:1 volume ratio. The HR-TEM analysis reveals that sorghum bran mediated iron nanoparticles were amorphous in nature with a diameter of 40–50 nm. The HR-TEM result also reveals that spherical iron nanoparticles were well dispersed and capped with water soluble hetero-cyclic components present in the sorghum extracts. Further, sorghum bran mediated iron nanoparticles used as catalyst for degradation of bromothymol blue.

***Plant peel extract:*** The facile green synthesis of magnetite nanoparticles was performed by using plantain peel extract. Venkateswarlu et al. [59] used waste plantain peel extract for reduction of iron salt to form Fe_3_O_4_ nanoparticles. Biomolecules present in the plantain peel extract was characterised by FTIR. The well dispersed spherical magnetic NPs (MNPs) sized below 50 nm were seen in a transmission electron microscopic image. The Brunauer–Emmett–Teller (BET) surface area of iron MNPs was 11.31 m^2^/g while higher saturation magnetisation was 15.8 emu/g. The obtained MNPs showed excellent magnetic behaviour and on the basis of BET surface area and pore volume results, the structure of nanoparticles was assigned to be mesoporous. By virtue of this property, as-synthesised nanoparticles can be used in the field of environmental remediation for the removal of toxic metals and dyes.

***Hydrothermal synthesis using plant extract:*** Ahmmad et al. [60] successfully synthesised highly pure hematite α-Fe_2_O_3_ nanoparticles by the hydrothermal synthesis method using green tea (*Camellia sinensis*) leaf extract. TEM images of hematite α-Fe_2_O_3_ showed nanoparticle spherical and highly porous particles with an average diameter of 60 nm. The surface area of the as-synthesised nanoparticles (22.5 m^2^/g) was four times higher, whereas the photocatalytic activity (capacity to generate OH radical when irradiated with visible light) was found to be about two times higher than commercially available hematite nanoparticles.

The photocatalytic activity of nanoparticle was assessed by measuring the amount of hydroxyl radical ions produced under irradiation of visible light. The as prepared α-Fe_2_O_3_ exhibited two time’s higher photocatalytic activity and better performance in a photo-electrochemical cell than commercial α-Fe_2_O_3_. Phumying et al. [61] synthesised Fe_3_O_4_ nanoparticles by the hydrothermal method using aloe vera plant extract and ferric acetylacetonate (Fe(C_5_H_8_O_2_)_3_). TEM revealed that synthesised nanoparticles were crystalline in nature having particle sizes of 6–30 nm. Morphology and chemical constituent was characterised by XRD and HR-TEM and results showed that synthesised Fe_3_O_4_ nanoparticles were inverse cubic spinel in structure without any phase impurities. Based on the coercivity, it was concluded that the nanoparticles were superparamagnetic in nature. The authors observed that increasing the reaction temperature and time resulted in magnetite nanoparticles with enhanced crystallinity and saturated magnetisation.

Table 3 summarizes the recent reports on synthesis of iron nanoparticles from plants and related materials.

### 2.5. Possible Mechanism of Nanoparticles Synthesis

Actual mechanism of nanoparticles synthesis by living organisms is not yet clear, however studies shows that enzymes produced from bacteria and fungi and biomolecules especially phenolic compounds in plant products cause the production of metallic iron nanoparticle [23,24,46]. In one study, Becerra et al. [20] utilised tannin powder a green reagent for synthesis of iron oxide NPs. Tannins consist of non-toxic polyphenolic compounds which act as reducing and stabilizing agents for the production of iron oxide NPs. According to them, most likely the presence of phenolic-OH groups and ortho-dihydroxyphenyl groups in chemical structure of tannins are involved in the formation of complexes with iron and also take part in redox reactions. In the formation of iron oxide NPs by tannins, the reactions undergo changes in electron structure. Tannins are oxidised to quinines and, by this reaction, iron salt is reduced to iron oxide nanoparticles.

Likewise, presence of biomolecules or combinations of chemically complex biomolecules, e.g., enzymes, amino acids, proteins, Vitamins, and polysaccharides, and organic acids such as citrates, may act as reducing and capping agents in nanoparticle synthesis [35]. The mechanism behind plant extract mediated metallic nanoparticle formation has not been clearly defined up until now. Not a single biomolecule of plant extract was involved in the fabrication of nanoparticles. Various plant components are rich in secondary metabolites and responsible for synthesis of metallic nanoparticles. Secondary metabolites include the polyphenols, flavonoids, tannic acid, terpenoids, ascorbic acids, carboxylic acids, aldehydes and amides. Many reducing sugars are commonly found in plants, and their presence is confirmed by the IR spectroscopic technique in different studies [62]. Phyto-chemicals in plant extracts possess ideal redox properties that allow efficient reduction of metal precursors for conversion into their corresponding metallic nanoparticles. In another study Becerra et al. [57] utilised the tannin of alfalfa. According to the assumption, tannins associated to alfalfa, derivate into radical tannins “R” causes reduction of metal under the influence of pH. The bioreduction process can be induced in the following way:

FeCl_3_ + H_2_O → [Fe(H_2_O)_n_]^3+^ + H_2_O (1)

R + [Fe(H_2_O)_n_]^3+^ + H_2_O → [Fe(H_2_O)_n_]^2+^ + H^+^ + R−OH (2)

(3) $${\left[\mathrm{Fe}{\left(H_{2}O\right)}_{n}\right]}^{2+}+2R^{-}+H^{+}+{\mathrm{OH}}^{-}\;\overset{\mathrm{pH}}{\to }\;{\mathrm{Fe}}^{2+}O^{2-}+{\mathrm{Fe}}^{2+}{\mathrm{Fe}}^{3+}O^{2-}+R-H+R-{\mathrm{OH}}^{-}$$

**Figure 2:**
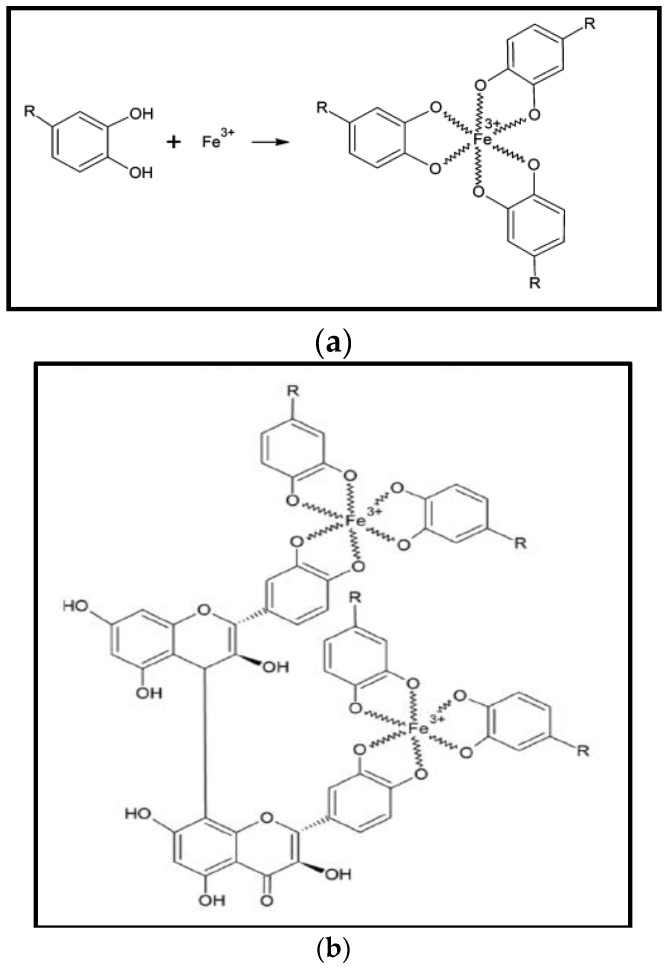
(**a**) Proposed chemical structure of Fe-P NPs [48]; and (**b**) proposed condensation mechanism of Fe-polyphenol [63].

In another study, Wang [44] proposed the iron-polyphenol complex nanoparticles (Fe-P NPs) structure, synthesised by *Eucalyptus* leaves. Reduction potential in *Eucalyptus* extract is due to polyphenols which make it able to reduce Fe^3+^ into Fe^2+^. However, extract does not completely reduce the Fe^2+^ to zero-valent iron. Fe^2+^ strongly stabilizes due to poylphenols ligands but rapidly oxidize in the presence of oxygen to give Fe^3+^-polyphenol complexes, this phenomenon commonly known as autoxidation. Thus, on reaction of iron metal solution with plants extract yields a black nano-iron colloid. A X-ray absorption (XAS) spectroscopy technique investigation suggested that plant polyphenols made chelate with ferric ion (Fe^3+^) and found in globular position (Figure 2a). Similar reaction mechanism was proposed for Sage (*Salvia officinalis*) mediated iron-polyphenol complex nanoparticles by Wang et al. [63]. Plant polyphenols and can be crosslinked by condensation of polyphenol on reaction between FeCl_3_ and plant polyphenol, as can be seen in Figure 2b.

## 3. Environmental Applications of Green Iron Nanoparticles

There are several green approaches to synthesize iron-based nanomaterials using different bio-chemicals and bio-reducing agents. Iron nanomaterials are significantly important for abatement of environmental pollution such as degradation of organic dyes, chlorinated organic pollutants and heavy metals removal, e.g., arsenic. Details about environmental applications of greener iron nanoparticles are as follows.

### 3.1. Degradation of Dyes

Hoag et al. [38] employed green tea (GT) synthesised iron nanoparticles to catalyse hydrogen peroxide for the degradation of the organic contaminant (bromothymol blue). The catalytic activity of green synthesised nanoscale zero-valent iron was more than that of Fe-EDTA and Fe-EDDS. From experiments, it was observed that by increasing concentrations of GT-nZVI, more hydrogen peroxide catalysed, which ultimately increased the degradation of bromothymol blue. Similarly, the reactivity of iron nanoparticles synthesised by aqueous sorghum bran extracts was tested for degradation of dye bromothymol blue by Njagi et al. [56]. In presence of iron nanoparticles and H_2_O_2_, Bromothymol blue degrades rapidly, demonstrating that the iron nanoparticles catalyses the reaction for production of free radicals from H_2_O_2_. The catalysis of H_2_O_2_ prompting the rate of reaction ultimately increases the rate of degradation of bromothymol blue [58].

In another report, green tea synthesised nZVI (Fe^0^) nanoparticles were employed for catalytic degradation of methylene blue (MB) and methyl orange (MO) dyes. The results indicate that the complete removal of methylene blue (MB) and methyl orange (MO) dyes from water was achieved at a concentration of 10–200 mg/L. As compared to MO, MB removed instantaneously as 80% of MB removed is first 5 min of reaction while 80% of MO dye removed after 1 h of reaction. Almost complete removal of the dyes was achieved after 200 min for MB and 350 min for MO, under the studied conditions. Green tea synthesised Fe nanoparticles proved to be more effective as a Fenton-like catalyst both in terms of kinetics and percentage removal compared to iron nanoparticles produced by borohydride reduction [39].

Huang et al. [62] used oolong tea extract for synthesis of iron nanoparticles (OT-Fe NP) further employed to degrade malachite green (MG). The results also showed that: first, polyphenols/caffeine in oolong tea extract acted as both reducing and capping agents in synthesis of Fe nanoparticles, leading to reduced aggregation and to increased reactivity of OT-Fe NP. Second, OT-Fe NP proved to be efficient in the degradation of MG, resulting in 75.5% of MG (50 mg/L) being removed. Kuang et al. [64] used extracts of three different teas i.e., green tea (GT), oolong tea (OT), and black tea (BT) separately for synthesis of iron nanoparticles. Synthesised iron nanoparticles were used as a catalyst for Fenton-like oxidation of monochlorobenzene (MCB). Highest degradation rate was achieved by green tea synthesised Fe NPs and was attributed to high polyphenol present in extract. Sixty-nine per cent degradation was observed for GT-Fe NPs while 53% by OT-Fe NPs and 39% by BT-Fe NPs in 180 min. Oxidative degradation mechanism was proposed for green tea synthesised iron nanoparticles and as follows.

At first, the MCB adsorbs onto the surface of Fe NPs and iron oxide formed in the Fe corrosion, as in Equations (4) and (5).

(1) Adsorption process:

MCB + Fe NPs → MCB/Fe NPs(4)

MCB + γ-Fe_2_O_3_ /Fe_3_O_4_ → MCB/γ-Fe_2_O_3_/Fe_3_O_4_ (5)

Fe^2+^ and Fe^3+^ leach from Fe^0^ and iron oxides on the surface of Fe NPs, as shown in Equations (6) and (7), and this process accelerates the decomposition of H_2_O_2_ and generates highly oxidative OH**·** radicals when Fe^2+^ was oxidised by H_2_O_2_ into Fe^3+^ (8)

(2) The process of generating hydroxyl radicals’ species:

Fe^0^ + H_2_O_2_ → Fe^2+^ + 2HO^−^ (6)

Fe_2_O_3_/Fe_3_O_4_ + H^+^ → Fe^2+^/Fe^3+^ + H_2_O (7)

Fe^2+^ + H_2_O_2_ → Fe^3+^ + HO^−^ + OH**·** (8)

In the meantime, generated Fe^2+^ and Fe^3+^ in the solution will react with H_2_O and yield oxyhydroxide Equation (9), which can also adsorb MCB. Furthermore, Fe^3+^ on the surface of Fe NPs was converted into Fe^2+^ and HO_2_**·** and the generated HO_2_**·** possibly further react with Fe^3+^ and favoured the decomposition of H_2_O_2_.

Fe^2+^/Fe^3+^ + H_2_O → FeOOH (9)

Fe^3+^ + H_2_O_2_ → Fe^2+^ + H^+^ + HO_2_**·** (10)

(3) Hydroxyl radicals’ species attack the MCB on the surface of Fe NPs:

MCB/Fe-NPs + OH**·**→ Reaction intermediates/Fe NPs (11)

Reaction intermediates/FeNPs + OH**·**→ CO_2_ + H_2_O (12)

Meanwhile, the generation of radical species rapidly reacts with the adsorbed MCB and also attacks MCB, resulting in mineralisation of some part of MCB on surface of Fe NPs into CO_2_ and H_2_O, which also involve in removal of COD (chemical oxygen demand). In reaction time of 180 min, the rate of dye degradation (81%) was high then and removal of COD (31%). The study illustrates that the complete mineralisation during Fenton-like process is not possible when COD content is high.

Iron ions polyphenol complex nanoparticles were effectively applied for degradation of dyes. Wang [44] employed stable colloidal iron-polyphenol complex nanoparticles (Fe-P NPs) mediated by eucalyptus for adsorption-flocculation against Acid black 194 dye was tested. It was observed that Acid black 194 adsorbed at 1.6 g per gram of Fe-P NPs at temperature 25 °C. Iron polyphenol nanoparticles (Fe-P NPs) mediated by three different plants i.e., *E. tereticornis*, *M. nesophila* and *R. officinalis* were compared for decolourisation of dye by Wang et al. [45]. About 100% of Acid black dye was decolourised, and 87% removal of total organic carbon (TOC) was achieved by Fe-P NPs. *E. tereticornis* Fe-P NPs showed good activity against dye degradation as compared to other nanoparticles and attributed to small size and good dispersibility of particles when analysed under SEM.

Huang et al. [65] studied the experimental factors such as the volume ratio of Fe^2+^ and tea extract, temperature, and pH to understand the influence of these factors on nanoparticles synthesis. Results show that there was a decline in the concentrations of Fe NPs with an increase in leaf extract because of decreasing Fe^2+^ concentration. Huang et al. further studied the reactivity of synthesised nanoparticles for degradation of dye, malachite green (MG). Degradation of MG by Fe NPs was influenced synthesised condition, pH whereas the high temperature also influenced on reactivity. In another study Luo et al. [46] utilised grape leaf extract mediated Fe NP for degradation of dye, acid Orange II. In this study it was found that the reactivity of plant mediated Fe NP was greater than the methanolic extract of grape leaves and Fe^2+^ solution both in water and methanol. Hence, the above studies show that the plant mediated iron nanoparticles were significantly effective for the degradation of various types of dyes under different experimental conditions. As compared to the conventional Fenton reaction, the Fenton-like reaction with plant mediated iron NPs takes place in a more sustainable manner. Plant mediated nanoparticles act as Fenton-catalyst with H_2_O_2_. Generally, oxidation depends on the activity of the hydroxyl radical (OH**·**) which produce in aqueous solution and due to reaction of Fe^2+^ and hydrogen peroxide, H_2_O_2_ (Equation (13)) as described below:

Fe^2+^ + H_2_O_2_ → Fe^3+^+ HO^−^ + OH**·** (13)

Fe^3+^ + H_2_O_2_ → Fe^2+^ + OOH**·** + H^+^ (14)

However, reaction pathway may be varying for different catalysts, or may depend on chemical nature of the catalyst as well as for the dye.

### 3.2. Removal of Heavy Metals

Rao et al. [50] used bio nanocomposite (phyto-mediated Fe^0^/Fe_3_O_4_ nanoparticles and yeast cells) to evaluate its capacity to remove hexavalent chromium which were proved to be good biosorbents. The sorption capacity of magnetically modified yeast cells was three times more than that of unmodified yeast cells. At the initial chromium concentration of 1000 mg/L and under optimum conditions, modified NCIM 3589 showed better adsorption capacity (186.32 mg/g) than modified NCIM 3590 (137.31 mg/g).

Madhavi et al. [66] reported a single-step synthesis of zero-valent iron nanoparticles (ZVNI) at room temperature using the *Euclaptus globules* leaf extract. The reaction for synthesis of iron nanoparticles was increased by adding more extract. FTIR spectroscopy provided the information about the vibrational state of adsorbed molecules and, hence, the nature of surface complexes. The phytogenic Fe^0^ nanoparticles (ZVNI) were further used for the adsorption of Cr(VI) metal. Adsorption parameters such as dose of adsorbent (ZVNI), initial concentration of Cr(VI) and kinetics were also studied by batch experiments. The highest adsorption efficiency of ZVNI was 98.1% at reaction time of 30 min, and dosage of ZVNI was 0.8 g/L. One occurrence of particular interest was that phyto-synthesised iron nanoparticles (ZVNI) were stabilised and remained in that state for up to two months after preparation. Likewise Savasari et al. [12] synthesised green ZVIN by ascorbic acid, which was employed for reduction of Cd(II) from aqueous and ascorbic acid synthesised nanoparticles proved to be stable and efficient.

In two different studies, Mystrioti et al. produced stable colloidal suspensions of nZVI coated with polyphenol of green tea and studied their chromium removal efficiency from groundwater as well as their transport characteristics through representative porous media [67,68]. The effectiveness of the resulting GT-nZVI suspension with diameter of 5–10 nm was evaluated for the removal of hexavalent chromium Cr(VI) from polluted groundwater flowing through the permeable soil bed. Green tea extract is characterised as a higher antioxidant compound due to presence of polyphenols. Polyphenols enriched green tea extract plays dual role in synthesis of nZVI, since they have capability to reduce ferric cations, meanwhile shield nZVI from being oxidised and agglomerated, functioning as capping agents. Column tests were performed at different flow rates in order to analyse the effect of contact time between the nZVI attached on porous media and the flow-over solution on reduction of Cr(VI). According to the results of the study, reduction and removal of Cr(VI) from the aqueous phase can be increased by increasing contact time. Leaching tests indicate that chromium in precipitated form is insoluble. In the tested soil material, the total amount of precipitated Cr was observed to be in the range between 280 and 890 mg/kg of soil, whereas the soluble Cr was less than 1.4 mg/kg of soil, which was most likely due to the presence of residual Cr(VI) solution in the porous soil. Nano zero-valent suspension is a very conducive to remediation of a contaminated aquifer, and the use of stable nanoparticles makes this technique successful [67]. Metals adsorbed on nanoparticles via redox reaction, co-precipitation or surface adsorption process [74,75]. The reactivity of iron nanoparticles based on different factors which ultimate influence on removal mechanism of, e.g. iron nanoparticles with variable oxidation states, possess different chemical characteristics as well as their mechanism of reaction with contaminants might be dissimilar as described by Tang and Lo [76].

Heterogeneous reduction reaction take place during in-situ remediation of chromium [77]. Heterogeneous reduction reaction that is followed by precipitation of reduced chromium as follow in below equations:

(15) $${\mathrm{CrO}}_{4\left(\mathrm{aq}\right)}^{2-}+{\mathrm{Fe}}_{\left(s\right)}^{0}+8H_{\left(\mathrm{aq}\right)}^{+}\;\to \;{\mathrm{Fe}}_{\left(\mathrm{aq}\right)}^{3+}+{\mathrm{Cr}}_{\left(\mathrm{aq}\right)}^{3+}+4H_{2}O_{\left(1\right)}$$

(16) $$\left(1-x\right){\mathrm{Fe}}_{\left(\mathrm{aq}\right)}^{3+}+\left(x\right){\mathrm{Cr}}_{\left(\mathrm{aq}\right)}^{3+}+2H_{2}O_{\left(l\right)}\;\to \;{\mathrm{Fe}}_{\left(1-x\right)}{\mathrm{Cr}}_{x}{\mathrm{OOH}}_{\left(s\right)}\;+3H_{\left(\mathrm{aq}\right)}^{+}$$

In another recent study, Xiao et al. [69] effectively employed plant mediated iron nanoparticles for removal of chromium, synthesised by various leaf extracts. Plant were selected on the basis of their reduction potential, i.e., selected from high to low antioxidant potential such as *S. jambos* (L.) Alston (SJA) extract with strong reducing ability, *Oolong tea* (OT) extract with moderate reducing ability and *A. moluccana* (L.) Willd (AMW) extract with weak reducing ability. The study shows that removal of chromium (VI) was consistent with reducing capacity of plants extracts. One millilitre of SJA-Fe NPs colloidal were able to remove 91.9% of the Cr(VI) in 5 min and 100% in 60 min. TEM image of the SJA-Fe NPs showed that NPs were spherical with diameter about to 5 nm and amorphous in nature when studied by XRD. However, this study lacks information on whether the removal of chromium of depends on reduction potential of plants or the size of nanoparticles produced by the extracts.

### 3.3. Wastewater Treatment

Chrysochoou et al. [70] investigated the attributes related to the transportation of iron nanoparticles synthesised with polyphenol enrich solution of green tea utilizing two granular media, refined silica sand, as well as sand-coated with aluminium hydroxide. The green tea nZVI (GT-nZVI) injection caused a rapid decline in the pH of effluent from 8.5 to 2 owing to the presence of residuary discharged Fe^3+^ in the solution along with corresponding hydrolysis reactions. The elevation in the redox potential from 150 mV to 550 mV was reported despite the fact that GT-nZVI holds reducing Fe^0^. This phenomenon is the characteristic feature related to the oxidation of polyphenols available in green tea. The elevation in redox potential can be an indicator of transport of GT-nZVI in the subsurface when used as an in situ reactant.

He et al. [8] employed starch mediated bimetallic Fe/Pd nanoparticles for the degradation of TCE (trichloroethylene). Results from this study demonstrated that the starched Fe nanoparticles showed considerably less agglomeration however, higher dechlorination power than those produced without a stabilizer. At dosage of 0.1 g/L of the starched nanoparticles were able to degrade 98% of TCE within 1 h in water. Wang et al. [71] employed biosynthesised iron nanoparticles for treatment of eutrophic wastewater. This study first synthesised iron nanoparticles through a one-step room-temperature biosynthetic route using eucalyptus leaf extracts. To the best of the author’s knowledge, this is the first study to report on green tea synthesised nanomaterial utilised for remediation of eutrophic wastewater. Synthesised polydispersed iron nanoparticles employed eucalyptus leaf extract obtained from its leaf litter. Due to the presence of different phytochemicals, each with varied reducing power in the extract form, the nanoparticles were polydispersed unlike the more common practice where nanoparticles are synthesised using a chemical reducing agent. For the first time, biologically synthesised nanoparticles were used for the treatment of eutrophic wastewater. After 21 days, percentage removal of total nitrogen, total phosphorus, and COD was 71.7%, 30.4%, and 84.5%, respectively. The reason for very low phosphorus removal was assigned to the absence of precipitating agents such as calcium, magnesium or aluminium.

In another study, Wang et al. [72] utilised the leaf extracts of green tea and eucalyptus separately for the formation of iron nanoparticles (Fe NPs) and employed for the efficient removal of nitrate from wastewater. Synthesis of spheroidal iron nanoparticles (Fe NPs) was confirmed by employing characterisation techniques. A comparison study was conducted between plant-synthesised and chemically-synthesised iron materials. Green tea and eucalyptus mediated Fe NPs were able to remove 59.7% and 41.4% of nitrate from waste water, respectively, compared to a 87.6% and 11.7% removal of nitrate by nZVI and Fe_3_O_4_ nanoparticles, respectively. Despite the higher removal efficiency of nZVI, the green synthesised Fe NPs were found to be more stable in nature. Reactivity of aged nZVI, green tea and eucalyptus synthesised Fe NPs was compared after being completely exposed to air for two months. Green tea and eucalyptus synthesised Fe NPs retained the same efficiency of 51.7% and 40.7%, respectively, whereas the efficacy of nZVI significantly dropped about 2.1-fold (45.4%).

### 3.4. Antibacterial Activity

Various studies confirm that iron nanoparticles possess good antimicrobial properties. The antibacterial effect of *Tridax Procumbens* synthesised iron oxide (Fe_3_O_4_) nanoparticles was investigated by Senthil and Ramesh [49] against gram negative bacteria *Pseudomonas aeruginos*. Kiruba Daniel et al. [73] used the leaf extract of *Dodonaea viscosa* for the synthesis of Cu, ZVI and Ag nanoparticles. The reduction of iron salt (ferric chloride) to ZVI nanoparticles was observed according to recorded instantaneous changes of reaction from yellow to greenish-black at room temperature. Iron zero-valent synthesised nanoparticles showed spherical morphology with an average particle size of 27 nm. The Fourier transfrom infrared (FTIR) study confirmed that the biomolecules in *D. viscosa* leaves such as flavonoids perform the reduction of metals salts, and their tannins, and saponins may act as capping agents. Capping of NPs with plant biomolecules prevent the oxidation of NPs to their oxide. Antimicrobial activity of biosynthesised NPs were evaluated against human pathogens viz. gram-negative bacteria *Escherichia coli*, *Klebsiella pneumonia*, *Pseudomonas fluorescens* and gram-positive bacteria *Staphylococcus aureus* and *Bacillus subtilis*. These biosynthesised NPs were proved as effective antimicrobial agents against specific human pathogens.

### 3.5. Stabilised/Immobilised Plant Mediated FeNPs for Degradation of Pollutants

Nanoparticles have the tendency to aggregate which can reduce the effectiveness of nanoparticles. This problem can be overcome by incorporating nanoparticles on any solid support such as polymers, zeolites, silica, etc. Literature shows that plant synthesized iron nanoparticles were successfully stabilised with different materials and used for pollution remediation (Table 4). Smuleac et al. [78] incorporated the Fe and bimetallic Fe/Pd nanoparticles in PVDF (polyvinylidene fluoride) membrane by green chemistry route. Green tea extract was used as a reducing agent for formation of Fe/Pd metallic nanoparticles in PVDF membranes modified by polyacrylic acid (PAA). PVDF/PAA membrane containing Fe nanoparticles was observed by SEM. An SEM image shows that the size of nanoparticles ranged from 20–30 nm with some aggregates between 80 and 100 nm. Further, reactivity of membrane supported nanoparticles was evaluated for the degradation of toxic organic pollutants known as trichloroethylene (TCE). Dechlorination of TCE was linearly increased with the increasing amount of iron (Fe) immobilised on the membrane. However, when catalytic Pd metal was added to form bimetallic Fe/Pd, the addition increased the degradation of TCE.

In another study, zerovalent iron (ZVI) nanoparticles with an average particle size of 59.08 ± 7.81 nm were synthesised by reaction of ferric nitrate with tea liquor. In addition, green zero-valent iron (ZVI) nanoparticles were stabilised on montmorillonite K10. Montmorillonite supported iron zero-valent nanoparticles were effectively employed for removal of arsenic as a heavy toxic metal. Ninety-nine per cent removal of arsenic As(III) was achieved in a reaction time of 30 min. from its solution at both low and high pH (2.75 and 11.1). Montmorillonite K10 alone removed less As(III), than the percentage of the tested montmorillonite supported nanoparticles under similar conditions of reaction [79].

Prasad et al. [80] studied the removal of arsenite(III) and arsenate(V) from aqueous solution using green synthesised iron nanoparticles. *Mentha spicata* L. synthesised iron nanoparticles showed absorption peaks at 360 and 430 nm confirmed by UV-Vis. Transmission electron microscope (TEM) results revealed that iron nanoparticles have core-shell structure and ranged from 20 to 45 nm in diameter. The planer reflection of selected area electron diffraction (SAED) and X-ray diffraction (XRD) analysis suggested that iron particles were crystalline and belonged to fcc (face centred cubic) type. FTIR study suggested that biomolecules or functional groups like N–H, C=O, C=C and C=N present in *M. spicata* extract were involved in particle formation. The efficiency of nanoparticles-chitosan composite for the removal of As(III) and As(V) was found to be 98.79% and 99.65%, respectively. The effect of extract ratio on formation of iron nanoparticles was studied by Martínez-Cabanas et al. [81]. Among chestnut tree (*Castanea sativa*), eucalyptus (*Eucalyptus globulus*), gorse (*Ulex europaeus*) and Pine (*Pinus pinaster*), eucalyptus was selected for synthesis of iron nanoparticles due to its high antioxidant property and availability. Different ratios of iron and extract were used for nanoparticles synthesis. Nanoparticles suspensions were mixed with chitosan separately and characteristics of chitosan beads were studied. Results of the study demonstrated that the iron/extract ratio not only effected on stability but also effected on magnetism behaviour of beads. However, TEM showed not any significant morphology and size difference. Chitosan encapsulated iron nanoparticles with good characteristics of stability were used for removal of arsenic by batch and column experiments for removal of arsenic. Regeneration of adsorbent suggested that green synthesised chitosan incorporated iron particles may work as an effective tool for elimination of arsenic from contaminated water.

## 4. Environmental Implications of Iron Nanoparticles

Despite the tremendous environmental applications of iron nanomaterials, they also present a risk when the environment comes into direct contact with these nanomaterials. Improper waste management from industries, leakage, and most important pollution remediation can cause harm—specifically in ground water and soil remediation where iron nanomaterial can transfer from one medium to another. Among the different species of iron nanoparticles, nano zero-valent iron (nZVI) is considered to be very reactive. Once zero-valent iron (Fe^0^) used as permeable reactive barriers for in situ treatment of ground water, it undergoes to transformation in presence of contaminants as well as to exposed environment. Presence of iron NPs in environment induces many toxic impacts to microorganisms and soil fauna, directly and indirectly significant for environment. Considerable toxicological impacts of iron NPs on soil microorganisms and changes in microbial biomass can be caused by induced stress of nanoiron [82,83]. Antisari et al. [82] evaluated the impacts of engineered nanoparticles on soil microbial mass and observed the change in microbial mass of soil. Moreover, transcriptional and proteomic stress responses to soil bacterium *Bacillus cereus* by nanosized zero-valent iron (nZVI) particles were observed by Fajardo et al. [84].

Auffan et al. [85] reported the relationship between oxidation state of iron nanoparticles and cytotoxicity. For this purpose, they compared the cytotoxic impacts of nZVI and iron oxide nanoparticles (magnetite and maghemite) towards gram negative bacteria *E. coli*. The toxicity of nZVI was found to be higher than the other iron oxide NPs. It was thought that the toxicity was associated with oxidation of iron nanoparticles which generated the oxidative stress from reactive oxygen species (ROS). ROS includes highly unstable superoxide radicals, hydroxyl radicals and freely diffusible and relatively long-lived hydrogen peroxide, which adsorb on the cell membrane and disrupt the functioning of cell. In another study Lee et al. [86] found that nZVI exhibited strong bactericidal activity under anaerobic conditions with a linear correlation between log inactivation of *E. coli* and nZVI dose. The toxicity of nZVI under oxygen saturated conditions was significantly lower than under deaerated conditions, thought to be related to oxidation and formed an iron oxide layer. This phenomenon was confirmed by study of Li et al. [87] which demonstrated that complete oxidation of nZVI in aerobic conditions almost eliminated bactericidal effects. In addition, Fe(II) was found to be more toxic under deaerated conditions, suggesting that released Fe(II) from nZVI contributes to toxicity. Likewise, nZVI triggered the substantial physical disruption of cell membranes, which led to cell inactivation by penetrating the cell membrane and causing physical damage or by enhancing the biocidal effects of Fe(II).

Age and surface modification influence on toxicity behaviour of nZVI. Phenrat et al. correlated the chemical and surface properties of nZVI with toxicity. In this study, Phenrat et al. used fresh nZVI, aged nZVI (>11 months), magnetite, and polyaspartate surface-modified (SM) nZVI to mammalian cells. It was found that particle properties such as “redox” activity, sedimentation rate, and agglomeration, generated morphological changes in neuron cells and cultured rodent microglia of mammalian cells. However, surface modified nZVI showed less toxicity because of reducing particles sedimentation which ultimately limited the particle exposure to the cells. Fresh nZVI showed remarkable impacts while aged nZVI exhibited insignificant morphological changes in mitochondrial and reduced ATP levels in neuron cells [88]. Chen et al. [89] evaluated the toxic effects of three different solutions containing carboxymethyl cellulose nZVI (CMC-nZVI), nFe_3_O_4_ and ferrous ion solution Fe(II)_aq_ by exposing to early life stages of medaka fish. The CMC-nZVI solution was found to be more toxic to embryos as compared to Fe(II)_aq_ and nFe_3_O_4_. CMC-nZVI solution was comprised of different oxidised forms of iron, generated from nZVI caused hypoxia, developmental toxicity and ROS oxidative stress in medaka embryos. It is believed that physicochemical properties of nZVI change in aqueous medium, such as chemical reactivity, particles aggregation, etc., which further influence the bioavailability or uptake of the nanoparticles and modify the toxicity behaviour of nZVI in fish.

The literature shows that the stabilised nanoparticles or capping agents does not helpful to reduce the iron oxide NPs toxicity. In one study Baumann et al. [90] functionalised the iron NPs with four different coatings: ascorbate (ASC-IONP), citrate (CIT-IONP), dextran (DEX-IONP), and polyvinylpyrrolidone (PVP-IONP) and evaluated their acute toxicity towards neonates of the water flea *Daphnia magna*. The highest immobilizing effect was recorded for ASC-IONP and DEX-IONP. In the presence of neonates, both ASC-IONP and DEX-IONP agglomerated or flocculated and adsorbed to the carapace and filtering apparatuses, induced high immobilisation. Lower immobilisation was found for CIT-IONP. Furthermore, incomplete ecdysis occurred at high concentrations of ASC-IOPN, DEX-IOPN, and CIT-IONP. PVP-IONP did not induce any negative effect, although high quantities were visibly ingested by the daphnids. PVP-IONP showed highest colloidal stability without any agglomeration, adsorption, or dissolution. It was thought that hydrodynamic diameter or the kind of stabilizing forces did not cause toxicity in daphnids, however the factors like colloidal stability and release of ions from the material, generated ROS in daphnids.

Green alga is an ecological indictor and represents aquatic ecosystem health. Toxicity of superparamagnetic iron oxide nanoparticles (SPION) has been investigated towards green algae *Chlorella vulgaris. C. vulgaris* cells were exposed with three iron oxides NPs suspensions with different chemical concentrations. SPION posed substantial toxicity, disrupted the photochemical activity of algal cells by inducing oxidative stress, and inhibiting the cell division [91].

Lethal effects of iron nanoparticles towards aquatic organisms have been documented by different researchers. Li et al. [92] investigated the effects of nZVI on antioxidant enzymatic activities and lipid peroxidation in Medaka (*Oryzias latipes*). Results showed that nZVI caused a disturbance in the oxidative defence system for embryos and adults, as well as oxidative damage in embryos with some observed effects at concentrations as low as 0.5 mg/L. Adult fish also showed antioxidant balance disruption although they were able to recover afterwards. Furthermore, histopathological changes and morphological alterations were observed in gills and intestine of adult fish. Remya et al. [93] evaluated the chronic toxicity effects of iron oxide (Fe_2_O_3_) nanoparticles (500 mg/L) on certain haematological, ionoregulatory and gill Na^+^/K^+^ ATPase activity of an Indian major carp, *Labeo rohita*. As compared to control groups, significant increase in haemoglobin (Hb) content, red blood cell (RBC) count and haematocrit (Ht) value was noticed. Fe_2_O_3_ nanoparticles also caused some variations in ionoregulation resulting in hyponatremia (Na^+^), hypochloremia (Cl^−^) and hypokalemia (K^+^). A biphasic trend in gill Na^+^/K^+^ ATPase activity was also noticed. Taze et al. [94] observed the oxidative responses of the mussel *Mytilus galloprovincialis* after exposure to iron oxide NPs and to iron oxide NPs incorporated into zeolite for 1, 3 and 7 days. Results showed that both effectors induced changes on animal physiology by causing oxidative stress in haemocytes of exposed mussels compared to control animals. Toxicity effects were observed by the significant increase in reactive oxygen species (ROS) production, lipid peroxidation, protein carbonylation, ubiquitin conjugates and DNA damage.

Blinova et al. [95] evaluated the toxicity of nanosized and bulk iron oxide nanoparticles on *D. magna.* No significant difference was observed in biological effects of both sized nanoparticles of magnetite. Although, iron oxide NPs induced very low toxicity (EC_50_ < 100 ppm) to *D. magna* and duck weed *Lemna minor* in the standard acute assays. It was observed that at acutely subtoxic magnetite concentrations (10 and 100 ppm), the number of neonates hatched from *D. magna* ephippia was decreased.

In addition to this, secondary environmental impacts of nano zero-valent iron (nZVI) have been investigated in soil organisms. Few studies reveal that iron NPs including nZVI and magnetic nanoparticles have positive effect on soil microbial community and facilitate the carbon and nitrogen cycling in soil. Iron oxide magnetic nanoparticles (IOMNPs) could potentially stimulate some bacterial growth and change the soil bacterial community structure, although bacterial abundance was not change. Meanwhile, soil urease and invertase activities significantly increased under IOMNPs amendment, which could be a consequence of the changes in the bacterial community [96]. El-Temsah and Joner [97] evaluated the ecotoxicological effects of nZVI coated with carboxymethyl cellulose on two species of earthworms, *Eisenia fetida* and *Lumbricus rubellus*. Earthworms were exposed to different nZVI concentrations ranging from 0 to 2000 mg nZVI kg·soil^−1^. Physical changes such as weight changes and mortality were observed for both species of earthworms at concentrations 500 mg·kg^−1^ soil. Reproduction was affected also at 100 mg nZVI·kg^−1^. However toxicity effects of aged soil nZVI were significantly reduced as compared to non-aged soils.

Fajardo et al. [98] studied the toxicity impacts of residual aged nZVI on metal contaminated soil. Heavy metal (Pb, Zn) polluted soils properties were evaluated after a leaching experiment. No negative effects on physico-chemical soil properties were observed after aged nZVI exposure. It was found that aged nZVI had negative effects on soil properties and NPs treatment increase Fe availability to soil. Moreover, toxic impacts of aged nZVI were related to metal contaminants of soil. However, Pb-nZVI soil showed changes in biodiversity, enhanced oxidative stress and Pb toxicity. Increased biological activity and decreased Zn toxicity were observed in Zn-nZVI soil. Canivet et al. [99] reported that iron nanoparticles had no significant cytotoxicity impacts on bryophytes (*Physcomitrella patens*). Similar results were observed in another study iron nanoparticles were exposed to seeds, iron nanoparticles could not show any detrimental impacts on seed germination at lower concentration of iron nanoparticles (0–5000 mg/L) [100]. Oxidative stress in plants and animal cells has been studied by many researchers. Studies show that many factors affect the behaviour of iron nanoparticles when they are released into the environment. Although the literature survey revealed that the presence of iron nanoparticles in soil exhibits less cytotoxicity in plants and can have positive impacts in plant germination, this scenario is not applicable to all environmental conditions due to the variation in soil types, concentration of iron nanoparticles used, and the chemical composition of NPs [99,100].

To combat the toxicological issues, research has advanced production of green nanomaterials, and studies have revealed that biosynthesised nanoparticles are less toxic than engineered nanoparticles [101,102]. In the case of biosynthesised iron nanoparticles, Nadagouda et al. [41] studied the toxic effects of phyto-synthesised nanomaterial on human keratinocyte cell. The biocompatibility of nZVI synthesised using green tea and borohydride as the reducing agent was assessed using methyl tetrazolium (MTS) and lactate dehydrogenase (LDH) assay by exposing cell lines to nZVIs for 24–48 h. LDH leakage increased with an increase in particle size, thereby stressing the cellular membrane. Hence, nZVI was synthesised using green tea since it is much smaller in size and has been shown to be nontoxic to human keratinocytes when compared to nanoparticles synthesised using the borohydride reduction process. Similarly, in another study Markova et al. [40] evaluated the impacts of plant-mediated iron nanoparticles on organisms have ecological importance including cyanobacterium (*Synechococcus nidulans*), green alga (*Pseudokirchneriella subcapitata*), and invertebrate organisms (*Daphnia magna*). The results of a toxicological assay showed a negative impact of green tea synthesised iron nanoparticles on cyanobacterium (*S. nidulans*), green alga (*P. subcapitata*), and invertebrate (*D. magna*) [103]. Above studies indicate that biosynthesised NPs are safe to environment and human beings, however in the literature, there is lack of reports concerning the toxicity of green synthesised iron oxide nanoparticles.

## 5. Conclusions and Future Perspective

This review focuses the production of iron nanomaterials via various green methods and their potential for remediation of environmental pollutants. The effort is made to highlight the various green agents for the synthesis of iron nanoparticles such as polymers, amino acids, bacteria, fungi, plant extracts, etc., and their reaction pathways to some extent. Moreover, this review discusses that particle size, morphology and other properties relates with the properties of materials, procedures and protocols. Literature shows that several plants and plant related materials have been exploiting for facile synthesis of iron nanoparticles, which proved to be good catalyst for widespread environmental application. Thus, plant materials look more feasible as agents for production of iron nanomaterials due to its environmentally friendly characteristics and economic value as an alternative to the large-scale production of nanoparticles. However, the mechanism has not yet been clearly described and there is need to explore the phytochemistry behind the synthesis of iron nanoparticles.

To achieve the sustainability of nanomaterial synthesis, more research is needed to explore more local and commonly available resources for the production of iron nanomaterials. Understanding the biochemical mechanisms involved in nanoparticle synthesis is a prerequisite to the success of any new methodology, and any solution must be economically competitive with conventional methods. Local resources should be utilised as their development will ultimately reduce the cost. In future research, more detailed study will provide a clear description of biomolecules and their role in mediating the synthesis of nanoparticles. The goal is to influence the rate of synthesis and improve nanoparticle stability. Moreover, research should be conducted to steer the production of iron nanoparticles toward increased reactivity to enhance environmental pollution degradation with minimum ecotoxicological impacts. In comparison to engineered nanoparticles, few studies confirm that biosynthesised nanoparticles are less toxic. In addition, a comprehensive risk assessment of green fabricated Fe NPs should be performed in which fate, transport, aggregation, dissolution and kinetics in processing of the nanoparticles is considered. In conclusion, green nanotechnology processes, as described in this paper, provide a strong foundation for the production of a variety of biochemical or functionalised nanoparticles that can serve as building blocks in the development of new products that can be applicable in environmental restoration sectors.

## Figures and Tables

**Table 1:** Size, morphology and environmental application of Fe^0^/Fe_2_O_3_/Fe_3_O_4_ nanoparticles synthesised by biocompatible green reagents.

Type of Nanoparticles	Biochemical Agents	Size and Morphology	Environmental Application	Reference
Stabilised bimetallic Fe/Pd nanoparticles	Starch	14.1 nm Discrete well dispersed	Degradation of chlorinated hydrocarbons in water	[8]
Fe_3_O_4_	Sodium alginate	27.2 nm Spherical	-	[9]
Fe_3_O_4_-polymer composite	Agar	50–200 nm Spherical and hexagonal	-	[10]
Fe noble metal nano-shell	Ascorbic acid (Vitamin C)	<100 nm Cubic	-	[11]
nZVI	Ascorbic acid	20 to 75 nm Spherical in chain	Cadmium (Cd) removal	[12]
Superparamagnetic Iron oxide(coating and functionalisation)	Ascorbic acid	5 nm (TEM) 30 nm (Hydrodynamic size)	-	[13]
Fe_3_O_4_	l-lysine amino acid	17.5 nm and spherical Crystalline		[14]
nZVI	l-glutamic acid, l-glutamine, l-arginine and l-cysteine	-	-	[15]
Fe NPs	Haemoglobin and myoglobin	2–5 nm Aggregates	-	[16]
Fe_3_O_4_	d-glucose gluconic acid	12.5 nm Roughly spherical Crystalline	-	[17]
Fe_3_O_4_	Glucose and gluconic acid	4–16 nmCrystalline	-	[18]
Carbon encapsulated iron NPs	Wood derived sugar	Nano-sphere 100–150 nm iron-core 10–25 nm	-	[19]
Iron oxide	Tannic acid	<10 nm	-	[20]
Fe-core shell structure	Chitosan-Gallic acid	~11 nm Cubic	-	[21]

**Table 2:** Size, morphology and environmental application of Fe^0^/Fe_2_O_3_/Fe_3_O_4_ nanoparticles synthesised by microorganisms.

Micro-Organisms	Species Name	Size	Env. Aps	References
Bacteria	*Actinobacter* sp.	10–40 nm cubic	-	[22]
	*Actinobacter* sp.	<50 nm	-	[23]
	*Thermoanaerobacter* sp.	~13 nm	-	[24]
	*Bacillus subtilis*	60–80 nm Spherical	-	[25]
	*Thiobacillus thioparus*	-	-	[26]
Fungi	*Fusarium oxysporum* and *Verticillium* sp.	20–50 nm Spherical	-	[27]
	*P. chlamydosporium*, *A. fumigates*, *A. wentii*, *C. lunata* and *C. globosum*	5–200 nm	-	[28]
	*Aspergillus*	50–200 nm	-	[29]
	*Alternaria alternate*	~9 nm	Antibacterial activity	[30]
Algae	*Sargassum muticum*	18 ± 4 nm cubic	-	[31]
	*Chlorococcum* sp.	20–50 nm Spherical	Reduction of chromium	[32]

**Table 3:** Size, morphology and environmental application of Fe^0^/Fe_2_O_3_/Fe_3_O_4_ nanoparticles synthesised by different parts of plants and plants material.

Plants	Part Used	Size and Morphology	Environmental Application	Reference
*Camellia sinensis*	Leaf	5–15 nm Spherical crystalline	Bromothymol blue degradation (organic contamination)	[38]
Green tea	Leaf	40–60 nm amorphous	Degradation of aqueous cationic and anionic dyes	[39]
Green tea	Leaf	70 nm–spherical crystalline	-	[40]
Tea	Tea powder	40–50 nm spherical	-	[41]
*Azadirachta indica*	Leaf	~100 nm	-	[43]
*Eucalyptus Tereticornis*	Leaf	40–60 nm Cubic	Adsorption of azo dyes	[44]
*Eucalyptus tereticornis*, *Melaleuca nesophila*, and *Rosemarinus officinalis*	Leaf	50–80 nm spherical	Catalyst for decolourisation of azo dyes	[45]
Grape	Leaf	15–100 nm quasi-spherical shape amorphous	Azo dyes such as acid Orange	[46]
*Carob*	Leaf	5–8 nm crystalline mono dispersed	-	[47]
*Azadirachta Indica*	Leaf	50–100 nm Spherical	-	[48]
*Tridax procumbens*	Leaf	80–100 nm crystalline irregular sphere shapes	Antibacterial	[49]
*Punica granatum*	Leaf	100–200 nm	Hexavalent chromium removal	[50]
*Hordeum vulgare* and *Rumex acetosa*	Leaf	10–40 nm amorphous	-	[51]
GarlicVine (*Mansoa alliacea*)	Leaf	13.82 nm–15.45 nm crystalline	-	[52]
*Terminalia chebula*	Fruit	<80 nm amorphous chain-like morphology	-	[53]
*Passiflora tripartitavar.*	Fruit	18.23–24.65 nm spherical crystalline	-	[54]
*Syzygium cumini*	Seed	9–20 nm spherical crystalline	-	[55]
Alfalfa	-	<5 nm		[56]
Alfalfa	-	1–10 nm		[57]
Sorghum	Bran	40–50 nm spherical amorphous	Degradation of bromothymol blue	[58]
Orange extract	Peel	30–50 nm crystalline cubic		[59]
Green tea	Leaf	40–80 nm crystalline	Photo catalytic activity	[60]
*Aloe vera*	-	6–30 nm cubic spinel structure crystalline	-	[61]
*Oolong tea*	Leaf	40–50 nm spherical	Degradation of malachite green	[62]
*Salvia officinalis*	Leaf	5–25 nm spherical	-	[63]
Green tea	Leaf	20–120 nm	Degradation of monochlorobenzene	[64]
Green tea	Leaf	70–80 nm spherical amorphous	Degradation of dye (malachite green)	[65]
*Eucalyptus globules*	Leaf	50 to 80 nm spherical	Adsorption of hexavalent chromium	[66]
Green tea	Leaf	5–10 nm Spherical	Removal of hexavalent chromium	[67]
Green tea	Leaf	-	Transport properties of nano zero-valent iron (nZVI) through soil	[68]
*S. jambos* (L.) *Oolong tea*, *A. moluccana* (L.), etc.	Leaf	-	Removal of chromium	[69]
Green-Tea	Leaf	-	Soil mineralogy	[70]
*Eucalyptus*	Leaf	20–80 nm amorphous	Treatment of eutrophic wastewater	[71]
*Green tea and eucalyptus*	Leaf	20–80 nm quasi-spherical	Nitrates removal	[72]
*Dodonaea viscose*	Leaf	50–60 nm Spherical	Antibacterial	[73]

**Table 4:** Polymer composite of phyto-synthesised iron nanoparticles for environmental remediation.

Plants	Part Used	Size and Morphology	Polymeric Support	Environmental Application	Reference
Green tea	Leaf	20–30 nm aggregates	Polyvinylidene fluoride (PVDF) membranes	Degradation of organic trichloroethylene (TCE) pollutant	[78]
Commercially available tea	-	48–70 nm Crystalline	Clay (montmorillonite)	Removal of arsenic	[79]
*Mentha spicata* L.	Leaf	20-45 nm poly dispersed cubic crystalline	Chitosan	Removal of arsenic	[80]
*Eucalyptus globulus*	Leaf	-	Chitosan	Removal of arsenic	[81]

## Abbreviations

BET	Brunauer–Emmett–Teller
COD	Chemical oxygen demand
EDS	Energy dispersive spectroscopy
EDDS	Ethylenediamine disuccinic acid
EDTA	Ethylenediaminetetraacetic acid
FTIR	Fourier transforminfrared
GC-MS	Gas chromatography-mass spectrometry
HAADF	Highly advanced techniques like the high angle annular dark field
HR-TEM	High resolution transmission electron microscope
LDH	Lactate dehydrogenase
MTS	Methyl tetrazolium
MCB	Monochlorobenzene
NPs	Nanoparticles
nZVI	Nanoscale zero-valent iron
PAA	Polyacrylic acid
PVDF	Polyvinylidene fluoride
SAED	Selected area electron diffraction
SCE	Saturated calomel electrode
SEM	Scanning electron microscope
TCE	Trichloroethylene
TEM	Transmission electron microscope
TOC	Total organic carbon
XAS	X-ray absorption spectroscopy technique
XRD	X-ray diffraction
ZVMI	Zero valent metallic iron
